# Prognostic Value of ANXA8 in Gastric Carcinoma

**DOI:** 10.7150/jca.40010

**Published:** 2020-03-15

**Authors:** Fangqi Ma, Xiaowei Li, Haiming Fang, Yueping Jin, Qin Sun, Xuejun Li

**Affiliations:** 1 The Graduate School, Anhui University of Traditional Chinese Medicine, Hefei, Anhui, China.; 2 Department of Gastroenterology, The Second Affiliated Hospital of Anhui University of Traditional Chinese Medicine, Hefei, Anhui, China.; 3 Department of Gastroenterology, The Second Affiliated Hospital of Anhui Medical University, Hefei, Anhui, China.

**Keywords:** Gastric carcinoma (GC), Annexin A8 (ANXA8), Biomarker, Independent prognostic factor

## Abstract

Gastric carcinoma (GC) remains one of the most common and deadly cancers worldwide. In China, the incidence and mortality rates related to GC were quite high. Annexin A8 (ANXA8) is a member of the annexins family of calcium-dependent membrane phospholipid binding proteins. According to recent research, the up-regulation of ANXA8 is closely associated with various types of tumors. However, the specific role of ANXA8 in GC remains unclear. In our study, we explored the prognostic value of ANXA8 in GC. Here, with the data from The Cancer Genome Atlas (TCGA) and Gene Expression Omnibus (GEO) datasets (GSE19826 and GSE13861) analyzed, we further performed quantitative real-time polymerase chain reaction (qRT-PCR) using 58 pairs of fresh-frozen tissues. We also subjected 152 pairs of formalin-fixed, paraffin-embedded GC tumor tissues from patients, and the adjacent normal gastric tissues (ANGTs) to immunohistochemical (IHC) analysis. Hence, we found an elevated expression of ANXA8 in tumor tissues with bioinformatics analyses, qRT-PCR, western blot and IHC. Over-expression of ANXA8 was strongly correlated with TNM stages and differentiation grades. Kaplan-Meier and cox proportional-hazard analyses showed that the increased expression of ANXA8 was strongly associated with overall survival (OS) and disease-free survival (DFS) in GC patients. Moreover, we found that ANXA8 is an independent prognostic factor of GC patients' OS and DFS. In brief, those results suggest that ANXA8 can act as an oncogene of GC development and can serve as a potential prognostic biomarker for GC treatment.

## Introduction

Gastric carcinoma (GC) is the world's leading cause of cancer-related death, especially among older males 1. Based on the latest GLOBOCAN 2018 data, GC is the 5th most common neoplasm and the 3rd most deadly cancer, with an estimated 783,000 deaths in 2018 2. In China, due to the Helicobacter pylori (Hp) infection, poor diet habits and the lack of early endoscopic screening techniques, the incidence and mortality rates of GC are still high, it has been one of the major malignant neoplasms threatening the health of Chinese residents over the years 3,4. At present, surgery is still the preferred method for the treatment of GC, to the delight of GC patients and their family, the 5-year survival rate of stage I GC patients can reach to 85%-95% with the early surgical resection 5. However, the early symptoms of GC are not obvious, with most cases of GC detected at an advanced stage and accompanied by lymph node infiltration and distant metastasis 1. What make it more terrible is for those advanced GC patients who have received radical surgery or effective chemoradiotherapy, the 5-year survival rate is still low (< 40%) with poor prognosis 4,6,7. Although some progresses have been made in recent years, the mechanisms of the occurrence, development and metastasis of GC have not been fully explored. Therefore, the further exploration of new biomarkers for GC is of great significance.

ANXA8 is a member belongs to the annexins family and it was firstly called VAC-beta because its structure and function are similar to the human vascular anticoagulant (VAC) 8. The annexins are a superfamily of Ca^2+^/phospholipid binding proteins that participate in the regulation of a major pathway for communication between the Ca^2+^ signaling and the Ca^2+^-regulated cell membrane dynamics in all eukaryotes, which closely attributed to various diseases, such as cancer, diabetes and autoimmune disorders 9-11. Since then, a number of studies have focused on ANXA8 and have demonstrated that ANXA8 has shown to be tightly associated with various types of malignant carcinoma. In 12-15, ANXA8 was found to be highly expressed in acute promyelocytic leukaemia (APL) cells. Meanwhile, ANXA8 has also been linked to the formation of endosomes and epidermal growth factor receptor (EGFR) turnover in Hela cells 16. Other reports demonstrated that the expression of ANXA8 is significantly increased in breast cancer 17-19, pancreatic cancer 20-22, ovarian cancer 23 and cholangiocarcinoma 24. Thus, ANXA8 is expected to be a new biomarker and molecular therapeutic target for the diagnosis, treatments and prognosis of multiple diseases, especially malignant tumors. However, the relevant mechanism of ANXA8 expression in GC has not yet been elucidated, nor has the functional role of ANXA8 been determined. Therefore, based on the results of previous studies, the prognostic value of ANXA8 in GC remains unclear.

In this study, we found a significant correlation between the raised expression of ANXA8 and GC. Moreover, ANXA8 is confirmed as an independent risk factor for overall survival (OS) and disease-free survival (DFS) of GC patients in our research. Taken together, these findings suggest that ANXA8 can serve as an effective prognostic biomarker in GC patients.

## Materials and methods

### High-throughput data processing

The expression data and corresponding clinical information for GC was obtained from The Cancer Genome Atlas (TCGA) (http://gdc.cancer.gov), Gene Expression Omnibus (GEO) GSE19826 and GSE13861 (http://www.ncbi.nlm.nih.gov/geo) datasets. All data were log2 transformed, and the results were analyzed by R and GraphPad Prism 7 software. The edgeR and limma package we used was based on the negative binomial distributions, empirical Bayes estimation, exact tests, generalized linear models (GLM) and quasi-likelihood tests. A logFC (fold change) ≥1.0 or logFC ≤-1.0 associated with a P value <0.05 was selected as statistically significant genes.

### Patients' information and tissues samples

All tissues samples were obtained from GC patients who underwent a gastrectomy at the Second Affiliated Hospital of Anhui University of Traditional Chinese Medicine (Hefei, China) and the Second Affiliated Hospital of Anhui Medical University (Hefei, China) between July 2013 and December 2014. None of them received radiotherapy or preoperative chemotherapy before surgery. All patients were followed up till December 2018. All specimens were handled and made anonymous according to the ethical and legal standards. All fresh tumor tissues specimens were snap-frozen in liquid nitrogen and stored at -80°C immediately after resection. Overall survival (OS) was defined as the period between surgery and death or the last contact. Disease-free survival (DFS) was defined as the period between surgery and any form of tumor recurrence.

### Western blot and antibodies

After denaturing by 10% SDS-PAGE, we transferred the total protein into nitrocellulose membranes. The membranes were settled in a series of steps with Tris-buffered saline containing 0.1% Tween-20 (TBST), and then, the targeted proteins were tested by the ECL (EMD Millipore, MA, USA) method. ANXA8 and α-tubulin antibodies for western blot were obtained from Abcam (ab111693) and Abcam (ab7291).

### Immunohistochemical staining and antibodies

Tissue specimens from 152 cases of GC fixed in the formalin and embedded in the paraffin (FFEP) for ANXA8 immunohistochemistry (IHC) staining. ANXA8 antibodies for IHC staining were obtained from Abcam (ab111693). After deparaffinization, hydration and blocking, the specimens were added into the primary anti-ANXA8 goat polyclonal antibody (diluted 1: 1000), and then incubated overnight at 4°C. Finally, all sections were assessed by comparison of staining between each gastric tumor and normal gastric specimen under microscopic. The scores were evaluation by two pairs: positive cells score and staining intensity score. The staining intensity score: 0: no staining; 1: slightly yellow than the background; 2: yellow brown; 3: brown. The positive cells score: 0: 0~5%; 1: 6~25%; 2: 26~50%; 3: 51~75%; 4: >75%. The IHC total score was calculated as positive cells score × staining intensity score. The total score was classified by four levels: 0 for the negative (-); 1-4 for the weak positive (+); 5-8 for the positive (++); and 9-12 for the strong positive (+++).

### RNA extraction and quantitative real-time polymerase chain reaction (qRT-PCR)

Total RNA was extracted with the trizol reagent (Invitrogen, NY, USA) according to the manufacturer's instructions. qRT-PCR was performed by the STBR green detection system qRT-PCR system (Takara, Japan) with the following primers for ANXA8:

sense primer: CTCCAGGTATGCA-CAGGCACACACAGGTGC;

anti-sense primer: CTCTTTCACCTCGGGGGCACCTTTCCCAGG.

GAPDH (forward primer: TGTGGGCATCAATGGATTTGG and reverse primer: ACACCATGTATTCCGGGTCAAT) (Servicebio Technology, Wuhan, China). GAPDH was used as reference control. The relative mRNA expression levels were quantified using the 2-ΔΔCt method. All qRT-PCR experiments were performed in triplicate.

### Statistical analysis

All data were analyzed with R. Differences between groups were compared by Student's t-test. The χ^2^ test was used to analyze the relationships between categorical variables. The Cox proportional hazards model was used to calculated survival rates. Survival curves were calculated by Kaplan-Meier method. P<0.05 was considered statistically significant from control.

## Results

### Overexpressed ANXA8 in GC tissues compared with ANGTs

**Figure 1:**
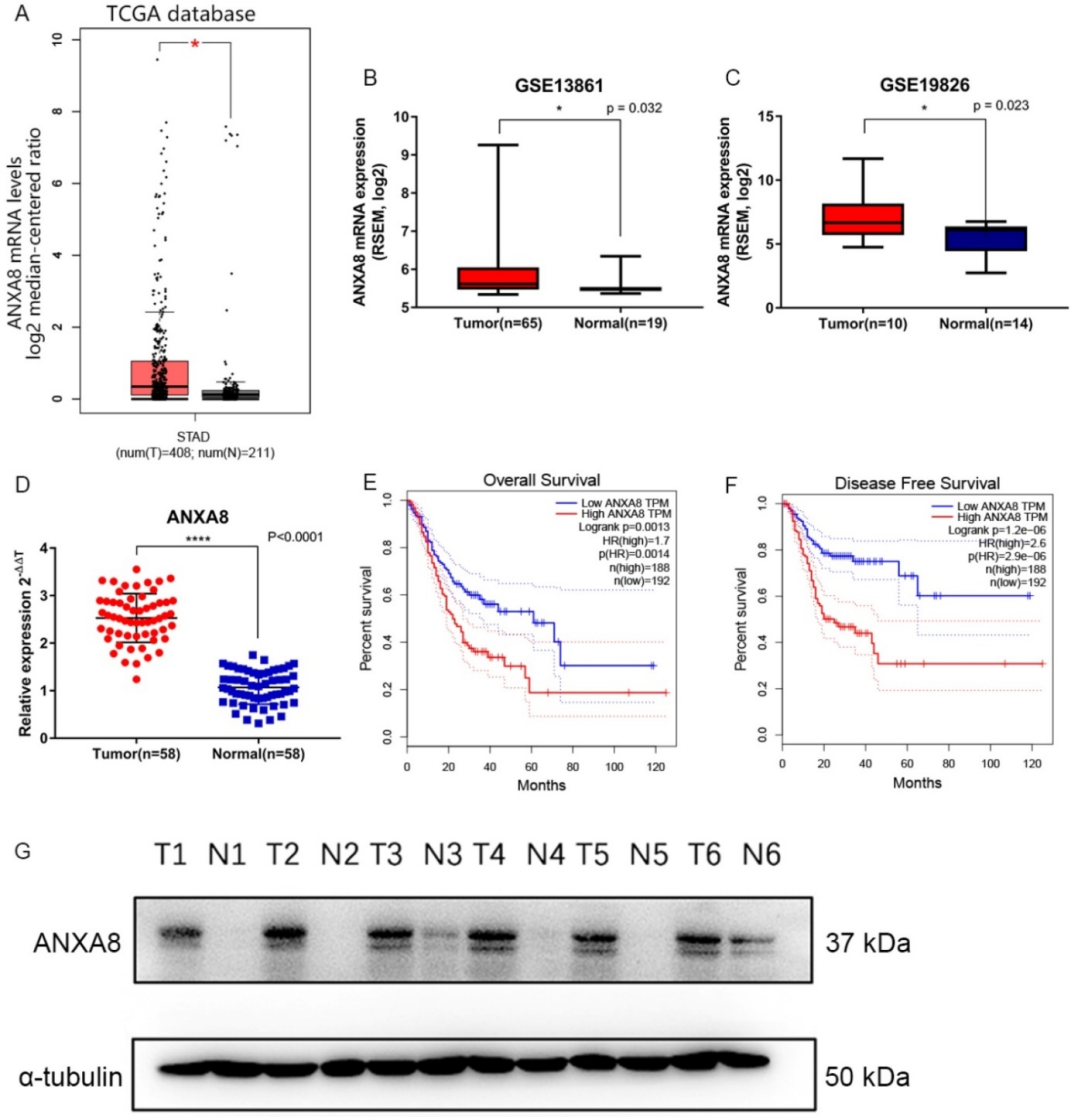
** (A)** ANXA8 is up-regulated in GC tissues in comparison with ANGTs in TCGA (tumor, n=408, normal, n=211, *P<0.05). **(B-C)** ANXA8 was highly expressed in GC tissues compared with normal gastric tissues in GEO (GSE13861, tumor, n=65, normal, n=19, *P=0.032; GSE19826, tumor, n=10, normal, n=14, *P=0.023). **(D)** The relative expression of qRT-PCR results between tumor (n=58) and normal (n=58), ****P<0.0001. All *P<0.05, **P<0.01, ***P<0.001, ****P<0.0001. Overall survival **(E)** and disease-free survival **(F)** curves for the GC patient groups with low (n=192) and high (n=188) ANXA8 expression. **(G)** Western blot results showed that the levels of ANXA8 were higher in six pairs of GC patients tumor tissues compared with ANGTs.

**Figure 2:**
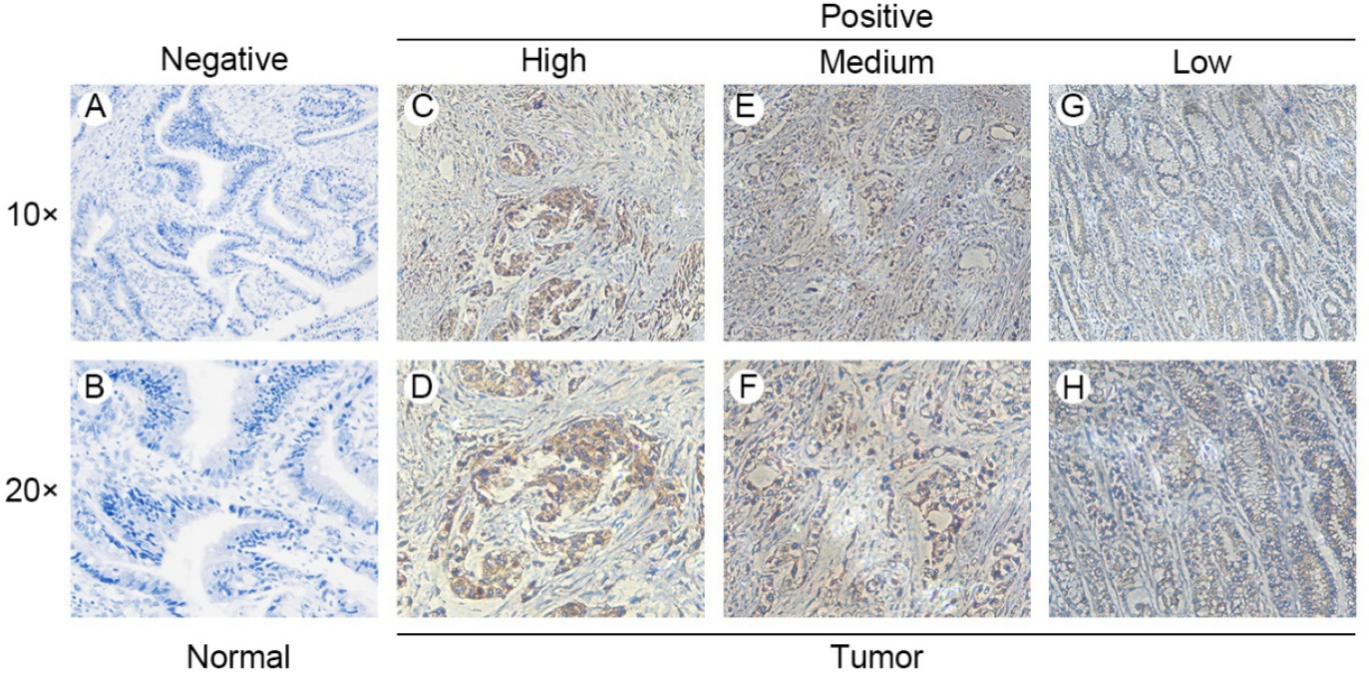
ANXA8 expression in GC tissues and ANGTs. IHC staining showed low ANXA8 expression in normal gastric tissues **(A and B)** and high **(C and D)**, medium **(E and F)** and low **(G and H)** GC tissues.

Gene expression data and corresponding clinical information were obtained from TCGA database and GEO datasets (GSE19826 and GSE13861). ANXA8 was found to be upregulated in all datasets and the detailed results were summarized in (**Figure 1A-C**). qRT-PCR in 58 pairs of fresh clinical samples verified the upregulation of ANXA8 in tumor tissues **(Figure 1D)**. IHC staining results showed that the expression levels of ANXA8 were high in 72 of the 152 gastric tumor cases and low in 64 (**Figure 2A-H**). Among six pairs of tissues, the results of western blot showed that the expression of ANXA8 was notably higher in GC patients tumor tissues compared with the ANGTs (**Figure 1G**). Taken together, ANXA8 was significantly upregulated in GC tissues compared with ANGTs.

### Correlations of ANXA8 expression with clinical parameters in GC

To explore the relationship between ANXA8 expression and GC clinicopathologic parameters, we analyzed the IHC staining results and corresponding clinical data in 152 pairs of GC tissues. The clinical characteristics were showed in **Table 1**. Our results indicated that ANXA8 expression is strongly related with TNM stage (P=0.002) and differentiation grade (P<0.001).

### Elevated expression of ANXA8 indicated poor prognosis in GC patients

**Figure 3:**
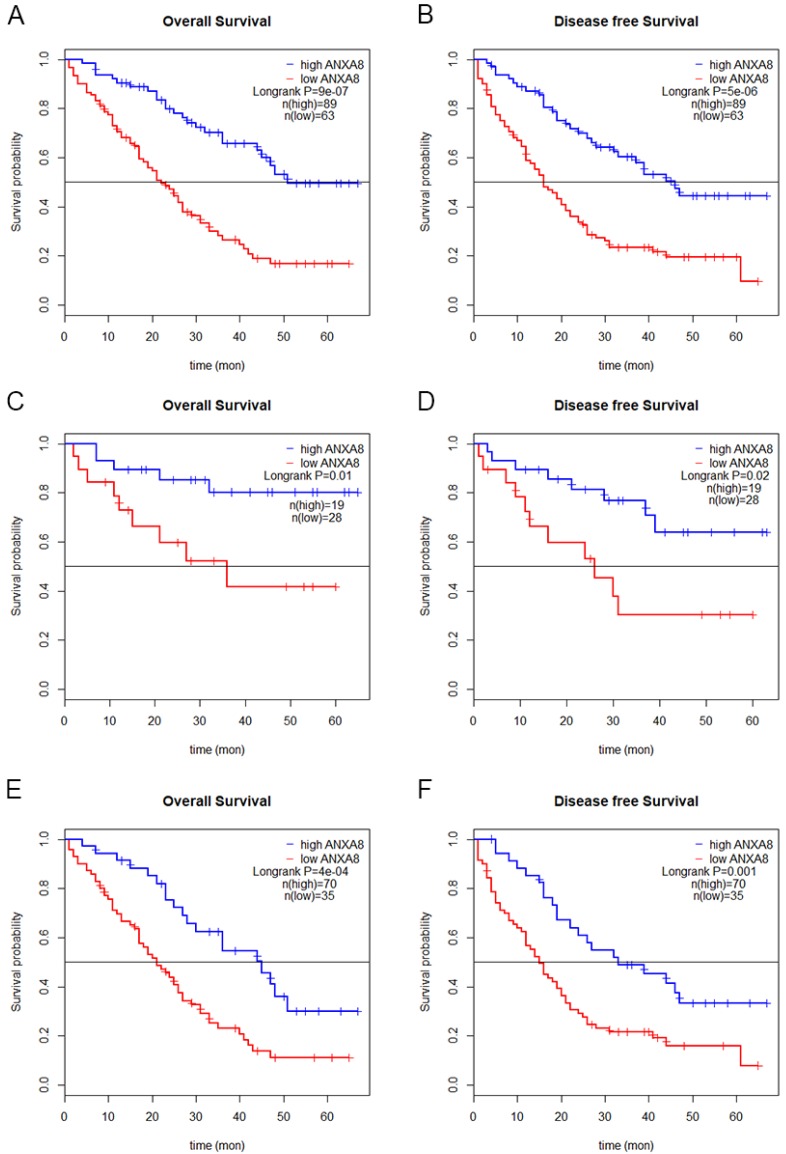
Overall survival **(A)** and disease-free survival **(B)** curves for the GC patient groups with low (n=63) and high (n=89) ANXA8 expression. A worse prognosis of high ANXA8 (n=19) expression compared with low ANXA8 (n=28) was observed in OS (p=0.01) and DFS (p=0.02) for early TNM stage** (C and D)**. Significant differences were found between high ANXA8 expression (n=70) and low ANXA8 (n=35) expression in OS (p<0.001) and DFS (p=0.001) for late TNM stage **(E and F)**.

To determine the prognostic value of ANXA8 in GC patients, we performed Kaplan-Meier (KM) analysis. KM curves showed that GC patients with high ANXA8 expression have worsen OS and DFS rates than GC patients with low ANXA8 expression in TCGA datasets (**Figure 1E,F**) and in 152 pairs of FFEP tissues (**Figure 3A,B**). Then we compared OS and DFS rates between early TNM stage and late TNM stage. A worse prognosis with high ANXA8 expression was observed in OS and DFS for early TNM stage (**Figure 3C,D**). Significantly differences were found between high ANXA8 expression and low ANXA8 expression in OS and DFS rates for late TNM stage (**Figure 3E,F**). According to these data analyses results, elevated expression of ANXA8 predicts a poor prognosis in patients with GC.

### ANXA8 serves as an independent prognostic marker in GC patients

We performed cox regression analysis to examine the role of ANXA8 in OS and DFS. Multivariate analysis indicated that ANXA8 expression (HR 2.293, 95% CI 1.317-3.991, p=0.003), lymph node metastasis (HR 3.572, 95% CI 2.047-6.233, p<0.001) and differentiation grade (HR 2.137, 95% CI 1.006-2.794, p=0.047) were associated with OS (**Table 2**). Furthermore, ANXA8 expression (HR 2.151, 95% CI 1.299-3.562, p=0.0029) and lymph node metastasis (HR 2.027, 95% CI 1.227-3.348, p=0.0058) were associated with DFS (**Table 3**). Taken together, ANXA8 can serve as an independent prognostic factor in patients with GC.

## Discussion

Annexins, which can be widely found in the eukaryotic cells both inside and outside, include a multigene family of phospholipid-binding proteins that are generally recognized as the regulator of the crucial signal pathway of ion (Ca^2+^) in membrane interactions. Meanwhile, diverse biological cellular activities involved signal transduction, cell growth, transformation, proliferation, differentiation and apoptosis 11, which are closely correlated with various membrane-related events organized by annexins. Therefore, those related disorders may cause a number of diseases, especially malignant tumors. However, ANXA8, the relative novel member of the annexins, has rarely been studied yet. The mechanisms of its functions in GC have not been explored and elaborated particularly.

Previous studies have shown that the expression of ANXA8 is elevated in various types of cancer tissues while limited in normal tissues. Early research on APL identified that over-expressed ANXA8 plays a significant role in the signal transduction pathway in the APL cells 12-15. It has been reported that ANXA8 expression is apparently upregulated in breast cancer, with a great relevance of tumor stages, grades and positive lymph nodes 17-19. Of note, ANXA8 is crucial in the maintenance of the late endosomal/lysosomal compartment physiological functions in cancer cells 16 and is able to interact with phosphatidylinositides and actin-associated membrane domains 25 which are closely related to cell migration, cytokinesis, vesicle transport and cellular immunity. It has also been clarified that high expression of ANXA8 in pancreatic cancer 20-22 and ovarian cancer 23 was correlated with tumor cell invasion and proliferation. ANXA8 transcription is down-regulated by EGFR and its downstream targets, phosphatidylinositol-3-kinase and Akt, are universally known as key factors of tumors proliferation, migration and metastasis 24. Recent studies have shown that ANXA8 regulates leukocyte recruitment to activate endothelial cells and affects CD63 and p-selectin delivery 26. Meanwhile, ANXA8 can organize the formation of CD63/VEGFR2/b1 integrin complex and over-activate VEGF-A signal transduction pathway 27. Furthermore, a previous study demonstrated that ANXA8 acts as a crux regulator of the endosomal regulation of cholesterol homeostasis, and this membrane binding is used to affect such a cytosolic regulatory protein dynamically 28.

Hence, ANXA8 may play a quite important and latent part in GC. However, no specific report has elaborated the relationship between the expression of ANXA8 and GC. Here, our group aimed to explore the relationship between GC and ANXA8. The existing studies on the clinical significance and biological role of ANXA8 have indicated that its function and mechanism in GC remains obscure. Our initiatory prediction based on TCGA and GEO datasets (GSE19826 and GSE13861) suggested that ANXA8 was tightly correlated with GC tumorigenesis and progression. To investigate the prognostic value of ANXA8 in GC, we analyzed the expression data and corresponding clinical parameters. Our results showed that ANXA8 is highly expressed in patients with poorer OS and DFS rates. Furthermore, we confirmed that significantly up-regulation of ANXA8 in GC was closely associated with worsen OS and DFS in TCGA datasets (**Figure 1E,F**). Then, we analyzed six pairs of samples between GC tissues and ANGTs by western blot, the results indicated that the expression of ANXA8 was remarkably higher in GC tissues (**Figure 1G**). Consistently, the IHC staining results of 152 pairs of clinical FFEP GC samples validated that the high expression of ANXA8 is positively related to lower OS and DFS rates (**Figure 3A,B**). Moreover, significant differences between high expressed ANXA8 and low expressed ANXA8 in late TNM stage of GC patients were discovered (**Figure 3E,F**), which showed a considerable importance of prognostic value for ANXA8 in GC patients. Meanwhile, multivariate cox regression analysis displayed that ANXA8 expression can be served as an independent prognostic factor in GC patients. Collectively, these findings demonstrate that ANXA8 shows a great potential as a novel biomarker for GC patients in the process of early diagnosis, treatment, clinicopathological parameters and prognosis.

In conclusion, we demonstrated that ANXA8 is notably elevated in GC tissues. Our data revealed that the high expression of ANXA8 represents poor prognosis for OS and DFS rates of GC patients and significantly correlated with TNM stages and differentiation grades. Accordingly, ANXA8 can serve as an independent novel prognostic factor for GC treatments. Further experimental validations are still required to clarify the molecular mechanisms by which specifically control and influence the process between ANXA8 and GC progression.

## Figures and Tables

**Table 1:** Correlation between ANXA8 expressions with clinicopathological characteristics of GC

Clinicopathological Variables	N	ANXA8 Expression		*P V*alue
		Low (63)	High (89)	
**Sex**				0.235
Male	95	42	53	
Female	57	21	36	
**Age, years**				0.374
<60	59	23	36	
≥60	93	40	53	
**CEA, μg/L**				0.505
<5	135	57	78	
≥5	17	6	11	
**HP**				0.188
Negative	60	28	32	
Positive	92	35	57	
**Lymph node metastasis**				0.111
N0, N1	53	26	27	
Presence	99	37	62	
**TNM stage**				**0.002**
Early (I & II)	47	28	19	
Late (III & IV)	105	35	70	
**Differentiation grade**				**<0.001**
Well	65	45	20	
Poor	87	18	69	

Abbreviations: CEA, carcinoembryonic antigen; HP, helicobacter pylori.

**Table 2:** Univariate and multivariate Cox regression analysis of risk factors associated with overall survival

Variables	Univariate analysis			Multivariate analysis		
	HR	95% CI	*P* Value	HR	95% CI	*P* Value
ANXA8 expression (High vs. Low)	3.100	1.928-4.985	**<0.001**	2.293	1.317-3.991	**0.003**
Sex (Male vs. Female)	1.004	0.646-1.561	0.985			
Age (≥60 vs. <60)	1.026	0.666-1.58	0.908			
HP (Positive vs. Negative)	1.011	0.657-1.554	0.962			
CEA (≥5 μg/L vs. <5 μg/L)	0.488	0.225-1.059	**0.069**			
Lymph node metastasis (N1,2 vs. N3,4)	3.572	2.047-6.233	**<0.001**	3.353	1.83-6.144	**<0.001**
TNM stage (Late vs. Early)	2.970	1.674-5.269	**<0.001**	1.517	0.797-2.888	0.205
Differentiation grade (Poor vs. Well)	2.137	1.362-3.351	**<0.001**	1.677	1.006-2.794	**0.047**

Abbreviations: CEA, carcinoembryonic antigen; HP, helicobacter pylori.

**Table 3:** Univariate and multivariate Cox regression analysis of risk factors associated with disease free survival

Variables	Univariate analysis			Multivariate analysis		
	HR	95% CI	*P* Value	HR	95% CI	*P* Value
ANXA8 expression (High vs. Low)	2.674	1.725-4.147	**<0.001**	2.151	1.299-3.562	**0.0029**
Sex (Male vs. Female)	0.753	0.502-1.131	0.172			
Age (≥60 vs. <60)	1.330	0.871-2.032	0.187			
HP (Positive vs. Negative)	0.849	0.568-1.27	0.426			
CEA (≥5 μg/L vs. <5 μg/L)	0.493	0.238-1.018	0.0559			
Lymph node metastasis (N1,2 vs. N3,4)	2.209	1.399-3.487	**<0.001**	2.027	1.227-3.348	**0.0058**
TNM stage (Late vs. Early)	2.268	1.373-3.748	**0.0014**	1.425	0.809-2.51	0.220
Differentiation grade (Poor vs. Well)	1.794	1.178-2.73	**<0.001**	1.393	0.868-2.234	0.169

Abbreviations: CEA, carcinoembryonic antigen; HP, helicobacter pylori.

## Abbreviations

ANXA8: Annexin A8
GC: gastric carcinoma
Hp: helicobacter pylori
VAC: human vascular anticoagulant
TCGA: The Cancer Genome Atlas
GEO: Gene Expression Omnibus
FFEP: fixed in the formalin and embedded in the paraffin
OS: overall survival
DFS: disease-free survival
TBST: Tris-buffered saline containing 0.1% Tween-20
qRT-PCR: quantitative real-time polymerase chain reaction
IHC: immunohistochemical
ANGTs: adjacent normal gastric tissues
APL: acute promyelocytic leukaemia
EGFR: epidermal growth factor receptor
EMT: the epithelial-to-mesenchymal transition
GLM: generalized linear models
CEA: carcinoembryonic antigen
